# Sociodemographic factors associated with the level of knowledge about management of epileptic patients in Peruvian dental students: a cross-sectional study under a multivariable analysis

**DOI:** 10.1186/s12903-023-02745-1

**Published:** 2023-01-30

**Authors:** Paolo Lurita-Córdova, Marysela Ladera-Castañeda, Flor Santander-Rengifo, Carlos López-Gurreonero, Alberto Cornejo-Pinto, Luis Cervantes-Ganoza, Antonieta Castro Pérez-Vargas, César Cayo-Rojas

**Affiliations:** 1 School of Stomatology, Universidad Privada San Juan Bautista, Av. Jose Antonio Lavalle Avenue s/n (Ex Hacienda Villa), Chorrillos, Lima Peru; 2 “Grupo de Investigación Salud y Bienestar Global”, Faculty of Dentistry and Postgraduate School, Universidad Nacional Federico Villarreal, Lima, Peru; 3 Academic Program of Dentistry, Universidad Peruana de Ciencias Aplicadas, Lima, Peru; 4 School of Stomatology, Universidad Científica del Sur, Lima, Peru; 5 Faculty of Stomatology, Universidad Inca Garcilaso de la Vega, Lima, Peru

**Keywords:** Knowledge, Odontology, Epilepsy, Students, Associated factors, Vulnerable patients

## Abstract

**Background:**

Epilepsy is a chronic neurological disease that could indirectly affect oral health, and it is necessary for dentists to be familiar with the specific needs of patients with epilepsy. Therefore, aim of the present study was to assess the factors associated with the level of knowledge about management of epileptic patients in Peruvian dental students.

**Methods:**

This analytical, observational, cross-sectional and prospective study assessed 312 dental students from a Peruvian university during February to April 2022. A validated questionnaire of 20 closed multiple-choice questions was used to measure the level of knowledge about dental management in epileptic patients. A logit model was used to assess the influence of variables: gender, age, year of study, marital status, place of origin and area of residence, with the level of knowledge in dental students considering a significance level of *p* < 0.05.

**Results:**

Of the total, 28.8%, 36.2% and 34.9% had a poor, fair and good level of knowledge, respectively, about the dental treatment of epileptic patients. On the other hand, it was observed that being a woman (OR = 0.44, CI 0.26–0.75) and being a third year student (OR = 0.39, CI 0.21–0.74) and fourth year student (OR = 0.43, CI 0.23–0.89) constituted a protective factor against poor knowledge about the dental management of epileptic patients, while being from the capital city constituted a risk factor. Finally, age, marital status and the students' area of residence were not considered influential factors (*p* > 0.05).

**Conclusion:**

Most of the students showed a poor and fair level of knowledge about the dental management of epileptic patients, with gender, academic year and place of origin being influential factors. It is advisable that authorities and teachers in the dental profession organize recurrent training programs on the care of patients with chronic diseases requiring special attention, since knowing general concepts, pharmacological management and dental care of epileptic patients will allow future dentists to develop competencies to improve and implement good quality care protocols for this group of patients.

## Background

One of the most common chronic neurological diseases is epilepsy, which is estimated to affect more than 70 million people worldwide [1–3]. In Latin America and the Caribbean, a prevalence range of 15.8–17.8 cases per 1000 inhabitants has been reported, and the incidence ranged from 77.7 to 190 cases per 100,000 inhabitants; and it is estimated that the prevalence of epilepsy in Peru is 20 per 1000 people [4, 5]. This disease affects the brain and is characterized by repetitive and unpredictable seizures in those affected [6, 7].

According to the World Health Organization (WHO) and the International League Against Epilepsy (ILAE), to diagnose epilepsy in a patient there must be chronic repetition of seizures, i.e. two or more spontaneous seizures [8, 9]. For epilepsy treatment, anticonvulsant drugs are usually prescribed to control seizures [6, 8, 10].

Epilepsy could indirectly affect people' oral health because the use of drugs to prevent this crisis could cause gingival hyperplasia and other alterations of the oral mucosa. In addition, dental fractures and/or soft tissue trauma are among the possible oral health consequences of epileptic seizures [11–13]. For this reason, some studies have reported that patients with epilepsy tend to have a poor oral health status compared to those without this condition [14, 15]. Among the characteristics associated with epileptic patients are the loss of dental pieces, dental fractures, high rate of dental caries and periodontal disease [16, 17].

In order to provide adequate treatment to patients with epilepsy, it is necessary for dentists to be familiar with specific needs of these patients, as well as to warn and prevent possible risks at dental level that could affect patients during an epileptic seizure [7, 18–20]. In addition, it is important for the dentist to be aware of the possible implications of prescribing certain drugs related to seizure control in order to prevent periodontal complications [21, 22].

In the dental management of epileptic patients, it is important that dentists can detail in the medical history: type of epilepsy, nature of the seizure, frequency, severity, triggering factors and medication the patient is receiving [23, 24]. The latter is of vital importance since there could be negative interactions with drugs normally used by the dentist when performing a surgical treatment [25]. Another point to take into account is that during the academic training of dentists, they should be taught the importance of making a consultation with the treating physician to find out the current state of the epileptic patient; plan the treatment days in advance in order to optimize the attention time and thus avoid an increase in stress that could cause a seizure [26]. In addition, the use of mouth openers with rubber wedges or teethers should be indicated during dental treatment in case a sudden crisis occurs, and thus avoid consequences due to the sudden closure of the jaw [27, 28]. To date (July 2022), very few studies have been conducted to assess the association between sociodemographic factors with the level of knowledge of dental students regarding the clinical management of epileptic patients. For example, Campos in 2017 and Guillen in 2020 reported that the students' level of knowledge on this topic was poor [29, 30].

Therefore, aim of the present study was to assess the sociodemographic factors associated with the level of knowledge about care management of epileptic patients in Lima and Ica, Peru; while the null hypothesis considered in this study was that there are no sociodemographic factors that are significantly associated with knowledge about the management of epileptic patients in these dental students.

## Methods

### Bioethical considerations

The present study respected the bioethical principles of the Declaration of Helsinki related to confidentiality, freedom, respect, and nonmaleficence [31]. We had the approval of an institutional ethics committee of the Universidad Privada San Juan Bautista whose resolution was No. 1523-2021-CIEI-UPSJB dated December 30, 2021. In addition, voluntary informed consent was requested on first page of the virtual questionnaire.

### Type of study

An analytical, observational, cross-sectional and prospective study was conducted. This manuscript was written according to the STrengthening the Reporting of OBservational studies in Epidemiology (STROBE) guidelines for observational studies [32].

### Population and selection of participants

The population consisted of 322 students of the professional career of Dentistry of the Universidad Privada San Juan Bautista (UPSJB), located in Lima and Ica, Peru. There were 121 students in their 3rd year of study, 111 students in their 4th year of study and 90 students in their 5th year of study. A statistical calculation of the sample size was not performed, since the entire population of Peruvian students from Lima and Ica who were in the third, fourth and fifth year of the professional career of Dentistry (N = 322) at the UPSJB, enrolled in the 2022-1 semester and who also gave their informed and voluntary consent, was included. Subsequently, students who did not complete the entire questionnaire were excluded, giving a total of 312 participants.

### Associated factors

The associated factors considered in the present study were the following variables: gender (X1), age (X2), academic year of study (X3), marital status (X4), place of origin (X5) and area of residence (X6) [11, 33].

### Elaboration of instrument

A questionnaire of 20 closed questions with polytomous answers (Yes/No/Don't know) was designed to assess dental students' knowledge about dental management of epileptic patients in three aspects: general knowledge about epilepsy with 6 questions (Q1–Q6), pharmacological knowledge about epileptic patients with 4 questions (Q7–Q10) and knowledge about dental care of epileptic patients with 10 questions (Q11–Q20). The overall level of knowledge was defined according to the following range: poor (0–10 points), fair (11–13 points) and good (14–20 points) [34, 35]. One point was awarded for each correct answer.

### Validation of instrument

The questionnaire content was validated through the judgment of three experts (MLC, LCG and CCR) in the research area with more than 15 years of experience in dental research and public health, who reviewed the conceptual definition and adapted the instrument to the research context. The test used was the validity coefficient (Aiken's V = 0.92; CI 0.65–0.99), which was acceptable. For construct validity, a confirmatory factor analysis was performed to define the dimensions and group the items, establishing three dimensions: general knowledge about epilepsy (Q1–Q6), pharmacological knowledge about epileptic patients (Q7–Q10) and knowledge about dental care for epileptic patients (Q11–Q20). Subsequently, the internal consistency reliability of the instrument was assessed by the Kuder–Richardson test (KR-20) and a score of 0.72 was obtained, proving to be acceptable. In addition, the questionnaire was administered at two different times with a span of 7 days to 30 dental students from the third to fifth year, randomly selected. To assess the correlation of scores, the order of the questions was altered to avoid recall bias. The correlation according to Pearson's R was very good (R = 0.96; CI 0.91–0.98).

### Procedure

The questionnaire was distributed to each student through their institutional and personal e-mails using the virtual program *Google Classroom*^®^. This was available online from February 15 until April 15, 2022. To avoid repetition of a student's participation, the survey form was configured to allow a single sending to the associated email address, in addition they were asked to place the initials of their name along with their age (for example: ACP43), to filter possible repetitions. The students' informed consent for participation in the study was at the beginning of the questionnaire, followed by the indications for completing it. However, they were free to refuse the assessment if they did not wish to complete it during its course. Only the principal researcher (PLC) had access to the students' personal data such as name, e-mail and telephone number. Only one submission per student was considered and each student was limited to a maximum of 10 min for completing the questionnaire. The study was conducted from February to April 2022. The assessment individual result was sent to the student who requested it by mail after all the study was completed.

### Statistical analysis

Data analysis was performed with the Statistical Package for the Social Sciences (SPSS) version 28.0. Descriptive statistics were applied to obtain frequency tables and bar graphs. Pearson's chi-square test was used for bivariate analysis and Fisher's exact test was used for expected values less than 5. Influencing factors were established with the logistic regression model (logit model) using odds ratio (OR). All analyses were performed, considering a significance level of 5% (*p* < 0.05).

## Results

The complete response rate of the participants was 96.89%, that is, 312 students (out of 322), of which 117 from 3rd year, 108 from 4th year and 87 from 5th year.

The average age of the participants was 25.7 ± 6.4 years. Female gender was the most frequent with 63.5% of all participants. The frequency of age group was similar between those aged 23 years or younger (50.3%) and those older than 23 years (49.7%). The highest percentage of students were in third year (37.5%). The vast majority of participants were unmarried (84.6%). Finally, 61.9% of dental students were from capital city and 91.0% lived in an urban area (Table 1).

**Table 1:** Sociodemographic characteristics of dental students from a Peruvian university

Variable	Category	Frequency	Percentage
Gender	Female	198	63.5
	Male	114	36.5
Age group	≤ 23 years	157	50.3
	> 23 years	155	49.7
Academic year of study	3rd year	117	37.5
	4th year	108	34.6
	5th year	87	27.9
Marital status	Unmarried	264	84.6
	Married or cohabiting	48	15.4
Place of origin	Capital	193	61.9
	Province	119	38.1
Area of residence	Urban	284	91.0
	Rural	28	9.0

Variable	Mean	Median	SD
Age	25.7	23.0	6.4

*SD* Standard deviation

Regarding knowledge of dental students about general aspects of epilepsy, a statistically significant association of gender with Q4 (A person who presents an epileptic seizure is necessarily an epileptic patient) was obtained (*p* = 0.022). Academic year was significantly associated with Q3 (Flashing lights or noises may be trigger factors for epilepsy) and Q5 (The epileptic patient may lose sphincter control during a seizure) (*p* < 0.001 and *p* = 0.018, respectively). Marital status was significantly associated with Q5 (*p* = 0.012). In addition, place of origin was significantly associated with Q2 (Congenital anomalies may be the etiology of epilepsy) and Q4 (*p* = 0.032 and *p* = 0.019, respectively). Finally, area of residence was significantly associated with Q3 and Q6 (Tobacco and alcohol may increase the possibility of an epileptic seizure) (*p* = 0.010 and *p* = 0.034, respectively) (Table 2).

**Table 2:** Knowledge of dental students from a Peruvian university about general aspects of epilepsy

Questions	Incorrect	Correct	Gender	Age group	Academic year of study	Marital status	Place of origin	Area of residence
	f (%)	f (%)	p*	p*	p*	p*	p*	p*
Q1. Epilepsy is a mental illness	221 (70.8)	91 (29.2)	0.332	0.959	0.287	0.490	0.107	0.424
Q2. Congenital anomalies may be the etiology of epilepsy	150 (48.1)	162 (51.9)	0.222	0.568	0.126	0.981	0.032	0.314
Q3. Flashing lights or noises may be trigger factors for epilepsy	109 (34.9)	203 (65.1)	0.434	0.499	< 0.001	0.800	0.108	0.010
Q4. A person who presents an epileptic seizure is necessarily an epileptic patient	165 (52.9)	147 (47.1)	0.022	0.254	0.859	0.411	0.019	0.056
Q5. The epileptic patient may lose sphincter control during a seizure	285 (91.3)	27 (8.7)	0.635	0.075	0.018	0.012^b^	0.681	0.080^b^
Q6. Tobacco and alcohol may increase the possibility of an epileptic seizure	87 (27.9)	225 (72.1)	0.059	0.286	0.509	0.628	0.062	0.034

*Based on Pearson's chi-square (*p* < 0.05, significant association); b: Based on Fisher's exact test (*p* < 0.05, significant association); *f* Frequency

Regarding knowledge of dental students about the pharmacological management of an epileptic patient, significant statistical associations of gender with Q7 (Carbamazepine and phenobarbital are antiepileptic drugs) were obtained (*p* = 0.042). Academic year of study was significantly associated with Q10 (Some anticonvulsant drugs may cause gingival recession) (*p* = 0.001). In addition, place of origin was significantly associated with Q7 and Q9 (Valproic acid may increase bleeding during surgery and delay healing) (*p* < 0.001 and *p* = 0.049, respectively). Finally, area of residence was significantly associated with Q8 (There are epilepsies that cannot be controlled with drugs) (*p* < 0.001) (Table 3).

**Table 3:** Knowledge of dental students from a Peruvian university about the pharmacological management of an epileptic patient

Questions	Incorrect	Correct	Gender	Age	Academic year of study	Marital status	Place of origin	Area of residence
	f (%)	f (%)	p*	p*	p*	p*	p*	p*
Q7. Carbamazepine and phenobarbital are antiepileptic drugs	78 (25.0)	234 (75.0)	0.042	0.845	0.481	0.147	< 0.001	0.647
Q8. There are epilepsies that cannot be controlled with drugs	288 (92.3)	24 (7.7)	0.587	0.191	0.103	0.554^b^	0.419	< 0.001^b^
Q9. Valproic acid may increase bleeding during surgery and delay healing	148 (47.4)	164 (52.6)	0.103	0.431	0.227	0.483	0.049	0.365
Q10. Some anticonvulsant drugs may cause gingival recession	209 (67.0)	103 (33.0)	0.031	0.496	0.001	0.778	0.859	0.172

*Based on Pearson's chi-square (*p* < 0.05, significant association); b: Based on Fisher's exact test (*p* < 0.05, significant association). *f* Frequency

Regarding knowledge of dental students about the dental care of an epileptic patient, significant statistical associations of gender with Q11 (During dental care of an epileptic patient, it is necessary to find out the last seizure date and frequency), Q15 (If an epileptic patient is medicated with valproic acid we should order a CBC test prior to dental care), Q16 (Local anesthetics generally do not cause complications in epileptic patients) and Q19 (After treatment of an epileptic patient it is necessary to recommend a follow-up with the treating physician) were obtained (*p* = 0. 018, *p* = 0.003, *p* = 0.005 and *p* = 0.013; respectively). Academic year of study was significantly associated with Q12 (Mouth opener or rubber wedges should not be used in dental care of an epileptic patient), Q13 (The epileptic patient should discontinue his or her medication prior to dental treatment), Q14 (A patient with epileptic seizure may suffer dentoalveolar avulsions and/or maxillary fractures), Q15, Q16, Q17 (If during dental care the epileptic seizure is repeated several times, the emergency department should be called) and Q20 (Status epilepticus is a neurological emergency that requires immediate attention) (*p* < 0. 001, *p* = 0.002, *p* = 0.003, *p* = 0.026, *p* = 0.032, *p* < 0.001 and *p* < 0.001; respectively). Marital status was significantly associated with Q13 and Q20 (*p* = 0.015 and *p* = 0.019; respectively). In addition, Place of origin was significantly associated with Q18 (If the patient has an epileptic seizure during dental treatment, the dental chair should be placed in the supine position) and Q19 (*p* = 0.049 and *p* < 0.001; respectively). Finally, area of residence was significantly associated with Q12, Q17, Q18 and Q20 (*p* < 0.001, *p* = 0.014, *p* < 0.001 and *p* = 0.013; respectively) (Table 4).

**Table 4:** Knowledge of dental students from a Peruvian university about the dental care of an epileptic patient

Questions	Incorrect	Correct	Gender	Age	Academic year	Marital status	Place of origin	Area of residence
	f (%)	f (%)	p*	p*	p*	p*	p*	p*
Q11. During dental care of an epileptic patient, it is necessary to find out the last seizure date and frequency	28 (9.0)	284 (91.0)	0.018	0.972	0.869	0.096^b^	0.275	0.091^b^
Q12. Mouth openers or rubber wedges should not be used in dental care of an epileptic patient	132 (42.3)	180 (57.7)	0.107	0.895	< 0.001	0.171	0.935	< 0.001
Q13. The epileptic patient should discontinue his or her medication prior to dental treatment	50 (16.0)	262 (84.0)	0.066	0.381	0.002	0.015	0.511	0.099^b^
Q14. A patient with epileptic seizure may suffer dentoalveolar avulsions and/or maxillary fractures	142 (45.5)	170 (54.5)	0.617	0.052	0.003	0.716	0.094	0.488
Q15. If an epileptic patient is medicated with valproic acid we should order a CBC test prior to dental care	42 (13.5)	270 (86.5)	0.003	0.536	0.026	0.112	0.738	0.240^b^
Q16. Local anesthetics generally do not cause complications in epileptic patients	91 (29.2)	221 (70.8)	0.005	0.655	0.032	0.167	0.085	0.611
Q17. If during dental care the epileptic seizure is repeated several times, the emergency department should be called	53 (17.0)	259 (83.0)	0.699	0.268	< 0.001	0.234	0.191	0.014^b^
Q18. If the patient has an epileptic seizure during dental treatment, the dental chair should be placed in the supine position	102 (32.7)	210 (67.3)	0.350	0.422	0.059	0.217	0.049	< 0.001
Q19. After treatment of an epileptic patient, it is necessary to recommend a follow-up with the treating physician	99 (31.7)	213 (68.3)	0.013	0.658	0.123	0.795	< 0.001	0.220
Q20. Status epilepticus is a neurological emergency that requires immediate attention	74 (23.7)	238 (76.3)	0.054	0.317	< 0.001	0.019	0.627	0.013

*Based on Pearson's chi-square (*p* < 0.05, significant association); b: Based on Fisher's exact test (*p* < 0.05, significant association). *f* Frequency

**Fig. 1:**
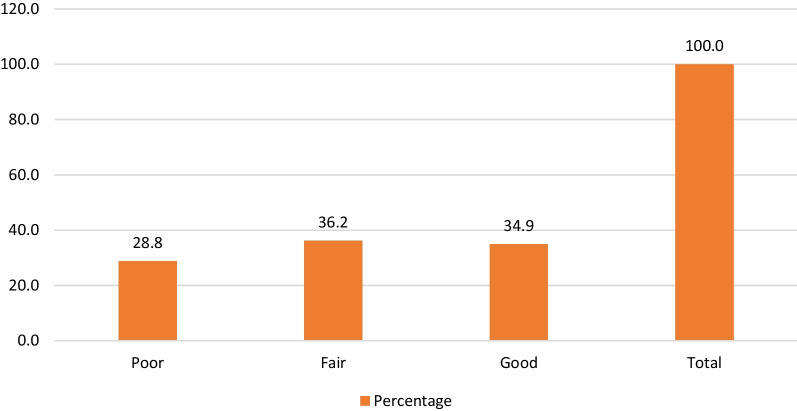
Knowledge level frequency of dental students form a Peruvian university about dental management of an epileptic patient

The 28.8% of the 312 dental students surveyed had poor level of knowledge about dental management of an epileptic patient, while 36.2% had fair level of knowledge, and finally 34.9% had good level of knowledge (Fig. 1).

On the other hand, according to bivariate analysis, it could be observed that the level of knowledge about dental management of epileptic patients was significantly associated with gender (*p* = 0.013), academic year of study (*p* = 0.012) and place of origin (*p* = 0.015) of dental students (Table 5).

**Table 5:** Knowledge level frequency of dental students form a Peruvian university about dental management of an epileptic patient

Variable	Category	Level of knowledge			p*
		Poor	Fair	Good	
		f (%)	f (%)	f (%)	
X1: Gender	Female	46 (14.7)	75 (24.0)	77 (24.7)	0.013
	Male	44 (14.1)	38 (12.2)	32 (10.3)	
X2: Age group	≤ 23 years	45 (14.4)	62 (19.9)	50 (16.0)	0.406
	> 23 years	45 (14.4)	51 (16.3)	59 (18.9)	
X3: Academic year of study	3rd year	26 (8.3)	43 (13.8)	48 (15.4)	0.012
	4th year	28 (9.0)	38 (12.2)	42 (13.5)	
	5th year	36 (11.5)	32 (10.3)	19 (6.1)	
X4: Marital status	Unmarried	80 (25.6)	95 (30.4)	89 (28.5)	0.363
	Married or cohabiting	10 (3.2)	18 (5.8)	20 (6.4)	
X5: Place of origin	Capital	65 (20.8)	71 (22.8)	57 (18.3)	0.015
	Province	25 (8.0)	42 (13.5)	52 (16.7)	
X6: Area of residence	Urban	80 (25.6)	100 (32.1)	104 (33.3)	0.138
	Rural	10 (3.2)	13 (4.2)	5 (1.6)	

*Based on Pearson's Chi-square, *p* < 0.05 (significant association). *f* Frequency

For multivariable analysis, the variables gender, age group, academic year of study, marital status, place of origin and area of residence were included, resulting significant in the crude model of logistic regression the female gender (*p* = 0.002), the third and fourth academic year of study (*p* = 0.005 and *p* = 0.020; respectively) and the capital as place of origin (*p* = 0.007). Thus, when adjusting the model, it could be observed that women were significantly 56% less likely to have poor knowledge about dental management of an epileptic patient than men (OR = 0.44, CI 0.26–0.75). In addition, dental students who were in third and fourth academic year of study were significantly 61% (OR = 0.39, CI 0.21–0.74) and 57% (OR = 0.43, CI 0.23–0.89) less likely to have poor knowledge, compared to students who were in fifth academic year of study. Finally, students whose place of origin was the capital city were 96% (OR = 1.96, CI 1.14–3.39) more likely to have poor knowledge about dental management of an epileptic patient than those whose place of origin was a Peruvian province (Table 6).

**Table 6:** Multivariable analysis of knowledge about dental management of an epileptic patient according to associated factors of dental students

Variable	Category	Crude model				Adjusted model*			
		OR	95% CI		*p*	OR	95% CI		*p*
			LL	UL			LL	UL	
X1: Gender	Female	0.42	0.24	0.72	0.002	0.44	0.26	0.75	0.002
	Male	Ref				Ref			
X2: Age group	≤ 23 years	1.32	0.72	2.39	0.367				
	> 23 years	Ref							
X3: Academic year of study	3rd year	0.38	0.19	0.75	0.005	0.39	0.21	0.74	0.004
	4th year	0.45	0.23	0.88	0.020	0.43	0.23	0.81	0.009
	5th year	Ref				Ref			
X4: Marital status	Unmarried	1.44	0.61	3.40	0.408				
	Married or cohabiting	Ref							
X5: Place of origin	Capital	2.21	1.25	3.91	0.007	1.96	1.14	3.39	0.016
	Province	Ref				Ref			
X6: Area of residence	Urban	0.63	0.26	1.54	0.312				
	Rural	Ref							

*Adjusted logit model for all variables that resulted with a *p*-value < 0.05 in the crude model; OR = Odds ratio, 95% CI = 95% confidence interval. For adjusted model of the knowledge of dental students about dental management of an epileptic patient, *p* < 0.001 (significant for the omnibus test of the model coefficient)

## Discussion

In this study it was found that 65% of dental students presented a level of knowledge between poor and fair. In addition, there were no statistically significant differences in the level of knowledge according to age, marital status and area of residence. However, the variables gender, academic year of study and place of origin significantly affected the level of knowledge about dental management of epileptic patients.

The predominantly fair level of knowledge about dental management of epileptic patients presented by dental students reaffirms the findings of Guillen [30], who also found that the majority of dental students in their fourth and fifth academic year presented fair levels of knowledge about dental management of epileptic patients. However, it should be noted that unlike Guillen's study [30], the present study included dental students in their third academic year and assessed other possible influencing factors such as gender, age group, marital status, place of origin and area of residence, since these have been considered in other studies that have assessed the level of knowledge in dental students [36–39]. It should also be noted that to date (July 2022), the present study is a pioneer in assessing the knowledge in this area considering six possible associated factors.

In the present study, the sociodemographic variables age group, marital status and area of residence did not significantly influence the level of knowledge presented by the students about dental management of epileptic patients, which is in agreement with the results of various studies that assessed other areas of knowledge in dental students, such as the one by Cayo et al., who reported that age and marital status did not influence the level of knowledge about metalloproteinases [36]. Likewise, the studies by Xu et al. about level of knowledge related to COVID-19 and by Abou el fadl et al. about level of knowledge about HIV patients reported that the variable area of residence was not significantly associated with the level of knowledge [40, 41].

In the statistical analysis of the present study, a multivariate logistic regression model (logit model) was included to identify the influential factor in the level of knowledge, showing that women were 56% less likely than men to have poor knowledge about the dental management of epileptic patients. This finding is probably due to the fact that women are more sensitive to situations where people are more vulnerable or require more personalized attention, bringing out their natural protective instinct to have a greater interest in training to treat this type of vulnerable patients [42, 43]. In addition, dental students in their third and fourth year of study were significantly (61% and 57%, respectively) less likely to have poor knowledge than students in their fifth year of study. This is probably due to the fact that in the university curriculum where the present study was carried out, there are courses prior to the last year of studies related to the dental management of patients with chronic diseases, which makes it easier for students to remember the theory learned than for those who are about to graduate [38]. Another factor to take into account is the updating of literature, since as time goes by, concepts, techniques, protocols and recommendations in the dental management of epileptic patients are renewed, which may allow students to find updated scientific information about social inclusion of vulnerable patients with each passing year [44, 45]. Finally, students whose place of origin was the capital city were 96% more likely to have poor knowledge about dental management of epileptic patients than those whose place of origin was a Peruvian province. This may be due to the fact that people from the province are generally more sensitive to people with illnesses or disabilities because of their culture or lifestyle. Because they are used to doing social work to help their fellow citizens, they may have paid special attention when training in the care of these patients [46].

The findings obtained in present study should be taken into consideration by the dental profession authorities and teachers in order to manage recurrent training about dental care for patients with chronic diseases requiring special assistance [47, 48], since knowing general concepts, pharmacological management and dental care for epileptic patients will allow future professionals to develop competencies to improve and implement good quality care protocols for this group of patients [49, 50]. It is also important for the student to know the possible implications of prescribing certain medications related to the control of epileptic seizures in order to prevent future periodontal complications [22, 24]. On the other hand, it is important that the dental student during his professional training takes into account and details in the clinical history the type of epilepsy, the nature of the seizure, the frequency, the severity, the triggering factors of the seizure and the medication that the patient receives, since there could be negative interactions with the drugs normally used by the dentist during a surgical treatment [20–22]. In addition, it is important to instruct the student to work in a multidisciplinary way, carrying out the interconsultation with the treating physician to know the current state of the disease. Likewise, the student must plan the treatment several days in advance and request laboratory tests if necessary [27, 28]. Due to the aforementioned, it is especially important to constantly train students in epileptic patient care, especially in countries like Peru, since the prevalence of epilepsy is higher in developing countries with tropical regions, since a One of the main causes of this disease in these countries is neurocysticercosis, caused by a helminth characteristic of tropical areas that affects the central nervous system, causing seizures with headaches [51, 52].

It is necessary to include various resources and/or pedagogical strategies within the curriculum as a transversal axis from an axiological approach so that the dental student can achieve procedural and attitudinal competencies that allow him to shape and develop his innate empathy [53], and consequently can provide quality service in his professional life to all types of patients, especially those who require special attention due to their physical limitations from chronic diseases of special care, as in the case of the epileptic patient.

## Future recommendations

It is recommended that more studies be conducted to assess the level of knowledge about dental management of epileptic patients, both at the undergraduate and postgraduate levels, in different populations, both nationally and internationally, without neglecting to evaluate sociodemographic variables under a multivariable statistical mode. In addition, it is also advisable to assess the level of knowledge about the management of epileptic patients in longitudinal studies, in order to identify whether the student's knowledge improves over time. In addition, it would also be advisable to repeat the same study with other public and private universities with the same number of years in the study plan and with similar curricula.

## Limitations

Among the limitations considered in the present study, we can mention that only dental students from the 3rd to 5th year of study were included, since students in the 1st and 2nd year of the professional career do not attend preclinical and clinical courses, in addition to not having contact with patients according to the curricular plan. In Peruvian universities it is common that from the 3rd year of studies onwards, dental students receive certain academic training that allows them to develop preclinical work with chronic disease patients, for example, when to make a medical interconsultation, management of adverse reactions to drugs and their stomatological implications, the correct filling of the medical history and its importance, knowledge about first aid, differences between urgencies and emergencies, among other topics. Another limitation of the present study was the lack of a criterion analysis for the instrument used, since to date (July 2022) there is no gold standard test that measures the level of knowledge in this subject. In addition, it should be recognized that the present study, having a cross-sectional design, does not allow an evaluation of the dynamism and sustainability over time of dental students' knowledge about the dental management of epileptic patients. It should be noted that the present type of study may have potential selection biases since dental students presented different sociodemographic characteristics. For this reason, possible confounding variables such as year of study, marital status, place of origin, and area of residence were controlled [54, 55]. On the other hand, the questionnaire was administered virtually asynchronously with a time limit of 10 min and without the possibility of repetition, to avoid greater bias [36].

## Conclusion

Most dental students showed a deficient level of knowledge about the dental management of epileptic patients. Being a woman and a third and fourth year academic student were protective factors against low knowledge, while being a student from the capital was a risk factor. In view of these findings, it would be advisable for the dental profession authorities and teachers to organize recurrent training in the care of patients with chronic diseases requiring special attention, since knowing general concepts, pharmacological management and dental care of epileptic patients will allow future dentists to develop competencies for improving and implementing good quality care protocols for this group of patients.

## Data Availability

The datasets used and/or analysed during the current study available from the corresponding author on reasonable request.

## Abbreviations

CI: Confidence interval
ILAE: International league against epilepsy
OR: Odds ratio
STROBE: Strengthening the reporting of observational studies in epidemiology
SPSS: Statistical package for the social sciences
WHO: World Health Organization
